# Risk-reducing surgery for breast and ovarian cancer risks - where are we now?

**DOI:** 10.1186/1897-4287-10-S2-A13

**Published:** 2012-04-12

**Authors:** N Hallowell, B Baylock, L Heiniger, M Price, P Butow

**Affiliations:** 1 Institute of Health & Society Newcastle University, UK; 2 kConFab Psychosocial Group on behalf of the kConFab Investigators - Centre for Medical Psychology and Evidence-based Decision-making (CeMPED), University of Sydney, Australia

## 

This talk will revisit some familiar issues about risk-reducing breast and ovarian surgery to manage cancer risks. I will focus upon the impact that surgery has on women’s lives and discuss their information needs. My talk will be illustrated with data collected during interviews with 40 Australian women as part of the kConFab Psychosocial study (Butow et al). Two emergent themes – *looking different* and *feeling different* - captured the psychosocial impact of surgery upon the interviewees. Many of the women said they felt differently about their bodies following RR surgery. All were relieved at having removed the risk of cancer that had previously been embodied in their breasts and ovaries, however reducing, risk by removing breasts and ovaries is not without costs. Interviewees reported experiencing a range of negative emotions and a series of unexpected bodily sensations following surgery and reflected upon positive and negative changes in their appearance. I will conclude that while women who undergo RR surgery are now informed about some of the *sequelae*, they are still not adequately prepared for the reality of undergoing this procedure. It will be suggested that, in addition to cosmetic outcomes, pre-surgical counselling needs to focus upon the experiential or sensational aspects of risk reducing surgery.

